# Mental Health Programme and Legislation in India: Some Observations and Experiences

**Published:** 2004

**Authors:** S. C. Malik

**Affiliations:** * Sr. Consultant, Sir Ganga Ram Hospital, New Delhi, Former Director Professor, Department of Psychiatry, Lady Hardinge Medical College & Hospital, New Delhi 110 001 and National Consultant, W.H.O. to the Ministry of Health, Govt. of India. satishsujata@hotmail.com


### Mental Health Programme & Legislation in India: Some Observations and Experiences

**S.C. Malik***

Dear Chairpersons, Mr. President, Esteemed Colleagues & Friends,

I am indeed overwhelmed by the honour you all have bestowed upon me for choosing me to deliver this prestigious oration - doubly so for also having elected me to the highest office of the president of the society about a decade ago. I have chosen to speak on mental health programmes & legislation and take you through a journey from post–independence period to the current scenario in terms of trials and tribulations which these programs underwent and I as a clinician, teacher and a member of the society witnessed, experienced, reacted and contributed to in my own humble way. I have divided my oration into four parts or periods—

1. 1947 - 1980: The Early Experiences
2. 1980 - 1995: Development and Formulation of the Mental Health Programme and Legislation
3. 1995 - 2000: Initiation of Implementation at National Level
4. The 21st Century: The Current Scenario

### 1. 1947—1980: The Early Experiences

It seems to me that the desire was not to be bound by the Indian Lunacy Act 1912, which was considered to be custodial in nature and out of date. The contribution of Dr. Vidya Sagar in terms of involving the family and to treat his patients in the makeshift tents outside the confines of the hospital (and thus outside the Act) and his humane approach is too well known. Development of General Hospital Psychiatric Units (GHPUs) in Calcutta and Bombay in 1930s & 1940s followed by Lucknow & Delhi soon after the independence is another example of the efforts to break the psychiatric practice free of these chains, imposed by the act as it were! In Wellingdon Hospital (now called the Dr. Ram Manohar Lohia Hospital), in early 60's I had the good fortune to head the GHPU and we soon ran into rough weather with our indoor facilities and strictly speaking it was illegal and any problems arising in these patients like absconding or even suicide attempt etc. had to be accounted for. With the help and intervention of the then Advisor in Mental Health, Dr. Erna Hoch, we framed the Admission and Discharge Rules and got them ratified from DGHS. These and many other such examples go to show that development of mental health services would have been seriously affected if the persons working in these setting were not innovative and courageous enough.

Soon after formation of the IPS in 1947, leadership within the society, in the field of enactment became evident and the first draft of mental heath bill was sent in 1950 to the Govt. of India. However there were number of reviews and revisions in the draft bill, which continued in 70's and 80's till both houses in the parliament passed it in 1987. The application of the MHA, 1987 and its implementation has brought about many other issues in the recent times, which I will come back to in the later part of the Oration.

### 2. 1980—1995: Development & Formulation of the Mental Health Programme and Legislation

In the meanwhile, during the 1960s & 1970s there had been a growing realization that there was a need for reaching out to the community for providing psychiatry services. Soon fervent activity in the form of epidemiological surveys in various parts of the country took place indicating the magnitude of the mental morbidity in general population thus blowing away the myth that psychiatric problems were confined to the affluent countries of the west. Infact, even rural areas were as prone as urban areas. Many pioneering efforts had been initiated by stalwarts like Dr. S.D Sharma at Baroda, Dr. N.N. Wig at Chandigarh and Dr. R.L. Kapur at Bangalore, which culminated in the development of the draft for the National Mental Health Programme in the early 1980s, in line with the then prevalent thought at the WHO Headquarters at Geneva for extending the mental health services to the unreached. Also projects done in the country (Raipur Rani in Haryana and Sakalwara in Karnataka) had shown the feasibility of integrating mental health care with general health care at the periphery.

I was again fortunate to have been involved in one of the two Workshops held in 1981 & 1982 for formulation of the National Mental Health Program which came into existence in August 1982 after being adopted by the Central Council

*Sr. Consultant, Sir Ganga Ram Hospital, New Delhi, Former Director Professor, Department of Psychiatry, Lady Hardinge Medical College & Hospital, New Delhi 110 001 and National Consultant, W.H.O. to the Ministry of Health, Govt. of India. Email: satishsujata@hotmail.com of Health-the highest health-policy making body in the country.

At the start, as per file records of DGHS/Min. of Health, the following gist of activities were considered essential or mandatory during the seventh plan:

1. Establishment of a National Advisory Committee on Mental Health.
2. Appointment of a suitable officer not below the rank of Asstt. Dir. General (ADG) for Mental Health along with his staff. Also the identification of a suitable officer at the Ministry of Health responsible for mental health activity.
3. Similar arrangements of an appropriate cell for mental health at State Directorate of Health with Program officers.
4. Training on Mental Health for at least one doctor at every district hospital during next five years. Coverage of at least 10% of PHCs for mental health services in next five years.
5. Establishment of new departments of Psychiatry at all medical colleges where they do not exist and strengthening of existing departments.
6. Establishment of certain task forces for (1) Operational aspects of mental health services (2) preparation of suitable manuals and health educational material (3) legislative changes for implementation of National plan on Mental Health.
7. Provision of at least three to four commonly used drugs at PHC levels and steps for the production of those drugs in the country in adequate quantity.
8. Inclusion of mental health knowledge and skills in the training of Health Staff in National Program of Health like Maternal and Child Health, I.C.D.S., Family Planning, Goitre and Blindness control. etc. for better implementation and success of these programs.

The National Advisory group of Mental Health was formed in August 1988 under the chairmanship of Secretary of Health and had two meetings –one in Nov. 88 at Nirman Bhavan and another in NIMHANS, Bangalore regarding achieving the various objectives in the plan document.

It was proposed to support Regional Centers at ten medical colleges in the country in various states. These centers were expected to co-ordinate the various mental health activities in the region and were to be involved in providing basic knowledge and skills to the PHC doctors and to paramedical workers.

Each center was given approx. Rs. 1.80 lakhs for this purpose on a yearly basis. However the feedback received from the states regarding proper utilization of funds was poor.

The achievements were

(a) The training manuals for doctors, paramedical staff and other educational material were developed with the help and co-ordinated effort of CIP, Ranchi, PGIMER, Chandigarh, and NIMHANS, Bangalore.

(b) Various organizations, state govt. agencies etc. were sensitized to mental health

(c) Training of trainers Programme conducted mainly at NIMHANS, Bangalore

The major difficulties encountered during the 7th plan were:

(a) Lack of resources

(b) Lack of clear-cut models to be adopted

It is worthwhile to mention here that Mental Health Act 1987 was passed during this period. However, it became operational only in April 1993

During the 8th plan, the allocation was raised to 200 lakhs from 100 lakhs.

The expenditure incurred under the NMHP for training in basic knowledge and skills in the field of mental health to the Primary Health Care physicians and paramedical personnel were as under-

1991-1992: Rs. 1.80 lakhs each to Medical colleges of Madras and Madurai (Tamil Nadu); K.G. Medical Colleges, Lucknow (U.P.); Regional Medical College, Imphal, Manipur. The total amount of Rs.7.20 lakhs.

1992-93: No expenditure was incurred

1993-94: An expenditure of Rs. 1.26 lakhs has been shown in the Demands For Grants 1995-96. Details not available.

1994-1995: NIMHANS, Bangalore (For conducting training program for PHC/Hospital doctors in Community Mental Health) - Rs.10 lakhs

**3. 1995 - 2000: Initiation of Implementation at National Level**

It was at this stage, in 1995, that for the first time National Consultant from WHO was inducted and a challenge thrown at me to take up this task requiring that something to be done urgently to revive and revitalize the program. It was conveyed to me that National Mental Health Program was not running and the year was about to end. There were prospects of the entire money meant for that year, remaining unspent. Could I do something? It was with a feeling of shock, indignation and surprise that I dug into the files, and learnt some of what has been described above.

I also realized that there was no mental heath policy or even National health policy document at the time of framing National Mental Health Program. The National Health Policy documents of 1983 as well as of 2002 (NHP-2002) make only a passing mention of mental health (see foot note).

NHP 1983– *"Special well co-ordinated program should be launched to provide mental health care as well as medical care and also the physical and social rehabilitation of those who are mentally retarded, deaf, dumb, blind, physically disabled, infirm and the aged."*

The recent National Health Policy document of 2002 (NHP-2002) is no better.

*"Mental health disorders are actually much more prevalent than is apparent on the surface. While such disorders do not contribute significantly to mortality, they have a serious bearing on the quality of life of the affected persons and their families. Sometimes based on religious faith, mental disorders are treated as spiritual affliction. This has led to the establishment of unlicensed mental institutions as an adjunct to religious institutions where reliance is placed on faith cure. Serious conditions of mental disorders require hospitalization and treatment under trained supervision. Mental health institutions are woefully deficient in physical infrastructure and trained manpower. NHP-2002 will address to these deficiencies in the public health sector."*

Perhaps, this lack of policy in mental health, might explain why the program failed. It also could not find acceptance by the medical community at large or with the health planners. Otherwise, it goes to the credit of the architects of the National Mental Health Program for having framed such a comprehensive document and getting it passed/ accepted by the central Council of Health.

A small group in the ministry of health was formed and we went into action immediately. The WHO/Min. of Health workshop in Feb. 1996 had endorsed the Central Council of Health resolution that National Mental Health Program be revived. It is to be noted that it was for the first time that these two meetings had stressed that Mental Health has assumed importance to be dealt with, as a major public health issue. Sartorius emphasizes that Public Health Problems are those that are frequent, grave in their consequences and exerting an impact on socio-economic development and that can produce immense disability and suffering and Mental Health Problems have always qualified for that. He further stresses that, as effective interventions are available through scientifically proven methods, thus qualifying to be dealt with through 'Primary Care' as per Alma Ata Declaration of 1978.

Whatever may be the argument, the planning commission, finance was not convinced about National Mental Health Program, going by our previous performance. The February 1996 workshop had on the other hand recommended extension of the program to 100 districts!

The immediate task was to see that the money allotted for the financial year does not lapse. Here, my earlier stint with the Ministry helped. Earlier, for sometime, I had been given the task of being Adviser to Govt. of India in Drug-Deaddiction Program wherein, we were able to set a norm for the training program for general duty doctors lasting for two to three weeks. We had successfully argued the case drawing similarity between IAS officers and medical doctors and had been also successful in de-centralizing the program bringing out from just the four centers of excellence, which had existed till then.

Applying the same norm that had already been developed, the contact was established with mental health institutes in Jaipur, Hyderabad, Guwahati, Chennai and Pune for running the training programmes. As, the manuals already existed, the task was simpler. In the program chalked out, there was provision to call the faculty from outside the state and lot of freedom was given to the course Directors in terms of augmenting or strengthening their departments with the unspent money by purchasing essential teaching equipment. Further, it was a great boost for these institutes as they could get recognition from their own states in terms of having participated in the National Program of Govt. of India. Thus the program, which hitherto was the monopoly of one or two institutes, had moved out and was soon to assume a truly national character because we planned it for as many states as we could reach. The argument that the training program will be utilized by very few in further training the others in the periphery due to their transfer, interest, etc. was also successfully tackled. There will be some change in the attitude of doctors after training in terms of stigma, correct referrals may result, and knowledge doesn't go waste any way—were the arguments. So enthusiastic were we in the Ministry/DGHS that this enthusiasm was communicated to those institutions now implementing it

Having established our credentials and earning some respect as we had utilized the entire funds for the year earmarked for the program, we went out to study how to extend the National program to other states at the level of districts.

Raipur Rani project had ended and now nothing existed there to be studied. A visit to Bellary district in Karnataka was made and also to Shankrapalli in Andhra Pradesh. NIMHANS, Bangalore developed the former and the latter by Institute of Mental Health, Hyderabad. After studying these models, we had discussions with those who were responsible for day-to-day running of the program and also with faculty of those institutions. I also had very useful discussion with Prof. N.N.Wig, one of the chief architects of the National Mental Health Program .A blue-print was thus made in terms of attaching a nodal institute to the district and giving it the responsibility of not only training the district doctors and other workers but also of seeing that the program was functioning properly. Another aspect was that the postgraduate trainees in Psychiatry in each state would thus be exposed to the community psychiatry concepts and through their posting see cases as they existed in the community. Wherever undergraduate teaching existed, this had the potential of being taught at that level and thus changing the negative mindset towards Psychiatry and Mental patients. Proper referral services could thus be established. Add to it the data collection and the research component and we had every reason to feel that the planners in the state and the teaching institutions will take it up and the program would grow.

Talking of data collection and research, I think it is an extremely important component but would lay great emphasis on providing services and continuity of these services even though pilot research project may have ended. This aspect should be considered and such a provision made otherwise the research project should not be started. I feel quite strongly about this.

The National Mental Health Program of 1982 is a very comprehensive document mentioning almost all the activities in metal health .It talks of treatment right from PHC to District level. Promotion, Prevention, and Rehabilitation sub programs are mentioned. It talks of improving teaching curriculum of Psychiatry in medical colleges, Carrying Training programs and Preparing Manuals at district level. This activity did take off but mainly at NIMHANS, Bangalore. It talks of linkages with other ministries, linkages of medical colleges and mental hospitals. It includes epilepsy, mental retardation and drug addiction in addition to mental illnesses. It talks of counselling services to mental retardation and genetically transmitted diseases. It has well defined aims and objectives. Approaches and targets have also been well defined. Promotion of positive mental health has also been stressed. Emphasis is on Primary Health Center. It even advises Indian system of Medicine to include mental health concepts at District level.

What went wrong then? Let us examine the causes closely so that we can learn from the mistakes.

1. Aims, Objectives, Approaches of National Mental Health Program: The language that is framed is very good but for health planners, bureaucrats, and non-psychiatrists we felt that it does not give a very concrete set of objectives so as to be easily picked up. They can be best be termed as Vision and Mission statements.

2. The National Advisory Body- the main body to oversee and monitor the implementation of the NMHP was very large and included high level officials from other ministries. It is impractical that all of them will ever meet and arrive at some meaningful conclusion. It is no exaggeration that they had only met twice since inception, that too soon after being formed and then forgotten. The fate of this body is not known.

3. Even though time frames were provided for targets to be achieved through detailed set of activities - how exactly these are to be achieved-it is unrealistic to expect the health planners and non psychiatrists to achieve these in the center and states.

4. Hence none of the activities took off to meet the targets to the extent that the Secretary (H), Govt. of India mentions on the file "this program is a non starter".

5. The program was being run mainly by NIMHANS, Bangalore and it must have been quite difficult to operate from there. It might have also resulted in generating jealousy and hostility in other institutes, which may have been impediments to the program.

The District Mental Health Program of 1996 had, on the other hand, the following set of objectives :

1. To provide sustainable basic mental health services to the community and to integrate these with other services. (This also included maintaining regular supply of some basic drugs).

2. Early detection and treatment of patients in the community itself.

3. To see that patients and their relatives do not have to travel long distances to go to hospitals or nursing homes in cities.

4. To take pressure off mental hospitals.

5. To reduce the stigma attached towards mental illness through change of attitude and public education

6. To treat and rehabilitate mental patients discharged from the mental hospital within the community. This step involved educating the family about mental illness and need for regular medication at home and strengthening the family support system.

7. To detect, manage and suitably refer cases of epilepsy and ensure availability of anti-epileptic drugs and others so as to reduce stigma towards epilepsy.

There was not much problem now in convincing the bureaucrats and planners in going about explaining these objectives as they were framed in a simple language and carried with them the air of desirability. Later on, we did encounter some difficulty towards inclusion of epilepsy as one of the objectives in the program as there were questions in the parliament that epilepsy could not be included amongst mental illnesses. Traditionally though, its detection and supply of anti-epileptic drugs in the community had been part of the National mental Health Program, wherever it was implemented. There was some lobby of neurologists also opposing its inclusion. So, seventh objective was dropped for sometime though at the level of implementation we continued the practice of looking after epileptics, as they and relatives would flock in these centers to get their anti-epileptic drugs. I think this practice continues till today.

The states were asked to identify one district and one nodal institution (like Medical college dept. of psychiatry) to be identified in their state. It was the task of this nodal institution to run training program and oversee the functioning of these centers wherein to and fro mutual referring system would start functioning benefiting both patients as well as staff who will be constantly learning from each other. Also the states were to be given an undertaking to continue these services by making provision in their state budget once the central scheme expires after 5 years. This was an essential part of the scheme if we had to get a sanction through the plan budget and that it was true of all national programs.

**Problems & Difficulties encountered were as under-**

1. As per Mental Health Act 1987, mental retardation was kept away from mental illness and all the matters pertaining to MR were to be referred to Ministry of Welfare (now social justice & empowerment). We were however involved through establishing linkages.

2. Similarly, as there was separate act for Drug Abuse and a separate Drug de-addiction program of the Ministry of Health/DGHS, we were to separate these activities from the program

3. Also, as there was a separate DISABILITY ACT OF 1995, the Rehabilitation efforts could be the responsibility of Ministry of Social Justice. Whereas the latter was well geared to taking up Mental Retardation, they were not eager to deal with Mental Disability- even though mental disability arising out of mental illness has been included as one of the disability as per the Act. Efforts are afoot to include the benefits to the mentally ill after the formulation of "IDEAS" –a measurement tool developed by IPS and its acceptance by the Govt. of India. However, it remains to be adequately tested and implemented

**Problems and Difficulties encountered by me were :**

1. There was no office for the consultant to sit and function. However, I used this as a way to cultivate friendship, establish understanding with staff in the DGHS/Minstry. There was no secretarial staff working under you so I was getting my typing work from outside and without getting any re-imbursement for it. Besides, I was taking my files home and working there myself prepared the SFS memo and learnt it from the concerned deputy secretary. I did not mind doing the job of even a peon, as insistence on such luxuries and indulgences would have jeopardized the entire effort and Programme. It was a good lesson to practice how not to involve your ego. Thus it became an opportunity for growth and a way of spiritual exercise for me.

2. Not being a govt. official can be a disadvantage as you are deprived of all the facilities, which come as a matter of fact manner to the govt. servant. However, I felt ' free' on the other hand to walk into Secretary/Minister's office not only in the center but also in the states where the DMHP was running. I could, with the persuasive and communication skills, which I had learnt from my superiors like Prof. R. M. Varma (former Director NIMHANS, Bangalore) successfully got the work done. I distinctly remember an instance when I had been able to get the Programme moving in Andhra Pradesh after personally meeting and convincing the State Health Minister.

**On the other hand, there were certain positive aspects of the experience.**

1. As the confidence in me of the Ministry grew, I was consulted in all matters pertaining to mental health. The DGHS /Min. sent me along with a senior official to advise Govt. of J&K regarding facilities to detenues in various camps in the state. The Secretary (H) took me along as a technical expert and advisor for visit to the mental hospitals in some of the states about which he had to give a report as per Supreme Court orders. In my present assignment, I was also asked to look after the Central Mental Health Authority as well. It was the follow up of visit to the mental hospitals that the Ministry/DGHS (under aegis of the WHO) and had a very important workshop about laying Minimum Standards of Care in these hospitals. Another offshoot of the Secretary's visit to these hospitals and sending the report was not the only improvement in them but also involvement of National Human Rights Commission in improvement of the state of affairs in the mental hospitals which I also attended and participated along with other officials in the Ministry. Later on NHRC initiated this as a project with NIMHANS, Bangalore.

2. Another important occurrence that happened was that WHO which had so far included Mental Health under the subhead 'Non Communicable Diseases' took it out and gave a separate enhanced budget under Mental Health. (It is noteworthy that there was a separate budget and program of Drug De-Addiction by the WHO). Prior to that the money not being utilized was being diverted to other programs of NCD. Not only there were WHO sponsored workshops and training and sensitization programs, we went about helping to build such departments of psychiatry in the country which lacked in such infrastructure as important books /library (separately for patients) and also professional books for doctors; essential equipment (like ECT machine, Boyle's Apparatus etc.) and drugs. This was particularly so in North Eastern States. We also provided vehicle for carrying out community work. We laid out norms for the equipment, vehicles etc. and tried to see that it is used by the psychiatrists and not diverted from the intended community work; at the same time not affecting the accountability. Meanwhile the DMHP was taking firm roots and spreading to other states so that when I left, it was successfully implemented in 22 states! (Presently in 27 districts in these states). The need and role of WHO consultant in all these matters pertaining to mental health was by now well accepted. This is particularly so as the expertise in mental health is otherwise lacking amongst the medical fraternity, not to talk of prejudices about psychiatry and mental health issues existing amongst doctors.

3. On the other hand, I have discovered that bureaucrats will listen to you if you leave your pre-conceived notions about their being inaccessible and biased. For example when I was in Lady Hardinge Medical College, I could persuade the Ministry to sanction De-Addiction Unit for the hospital; even though it meant admitting male patients, which happened for the first time in the history of this institution. It is history now that our move led to allowing male patients admitted in the entire hospital. We, psychiatrists, should be proud to acquire and judicially use communication skills. Doesn't Gita teach us that you simply do your duty and the consequences will be taken care of if your intentions have been good and unselfish? My assignment in the Ministry confirmed my views. Another thing that I have learned over the years is that it is professionalism that should be achieved and respected in others and that you should not stand aloof on matters of age or so. Very frankly I continue to learn from my students even today.

### 4. The 21st century: The Current Scenario

The National Mental Health Program should be rewritten and reframed after learning from the mistakes that we made in the past in terms of its improper implementation. We are in the 21st century and we have to take into account that Mental Health Act is in force and that there is a mental health authority at the center as well as at the state level to oversee its implementation and also in setting up proper services. Also another change is that there is law regarding Drug Abuse and also a separate program regarding the same. Consumer Protection Act has also been in vogue. All these realities will have to kept in mind. In 1982, when the NMHP was conceived and became operational, this was not so.

Also WHO has shifted its emphasis from prevalence rates to the concept of DALY—Disability adjusted life years and the concept of Quality of life (World Bank Report). Neuro-Psychiatric Disorders rank very high on the list of Global Burden Of Disease, when all diseases are ranked in terms of DALY of all ages and both sexes. As per latest reports available, unipolar major depression ranks fourth, even higher than ischaemic heart disease which ranks fifth. The projected rank in the year 2020 is rank second next to ischaemic heart disease.

Thus, mental disorders according to the above concept constitute about 8.1% more than the disability caused by many well-recognized disorders like cancer, (5.8%) or heart disease (4.4%). Apart from mental disorders, behavior related diseases e.g. diarrhoeal disease, accidents, malnutrition, AIDS, violence etc. which are not strictly mental disorders, but in the causation of which human behaviour is a significant factor constituting 34% of disability adjusted life years

We are living in an era of Information and Technology in which India is a leader. We must continuously update information and data collection at the peripheral level and judicially use it so as to evolve proper services. Both the formulation and evaluation of mental health policy require the existence of a well functioning and co-coordinated information system for measuring a minimum number of mental health indicators. Currently there is no system for annual reporting of mental health data. There is a need to invest resources in developing information monitoring systems which incorporate indicators for the major demographic and socioeconomic determinants of mental health, the mental health status of the general population and those in treatment (diagnosis, age, sex etc.). Further information regarding burden associated with disability (DALY) data is lacking in our country, the extent of burden on caregivers and the financial costs involved. This is very important for policy planning.

Even though there is no substitute to introducing the proper training and managing of mental illnesses at the undergraduate level, we should not do away with the training programs of doctors and other workers as has been lately done. Much work has been done in this direction and much experience gained, which should be further refined and applied. Infact, we can introduce giving CME credit points as is done in the advanced countries of the west and USA.

Research should form an integral component of all these programs. ICMR has always performed a leading role all through. Recently ICMR constituted a core committee in mental health (I am lucky to be a member) and produced a 'Vision Document' for the 21st century. It has been pointed out that the previous research experience gained through various ICMR projects (e.g. ICMR-DST project on severe mental morbidity) was not linked and utilized in the ongoing district mental health programme. If you know now how the district programme was evolved, I am sure one can understand the lapse which now can be corrected in further planning.

The 21st century has started on a very promising note, at least at the international level. After laying emphasis on high contribution of mental disorders towards disability and burden in the community through its formulation of the concept of DALY in 1993; there have been a series of other events.

(a) There has been the publication of review of the mental health needs of the developing countries and low-income countries by the Harvard Public School Report. (World Mental Health in 1995); as well as the publication of the Surgeon General's Report on Mental Health in December 1999, Child Mental Health in Jan. 2001 and Suicide Prevention in May 2001. The Institute of Medicine (IOM) Report on Neurological, Psychiatric and Developmental Disorders in Developing Countries, meeting the challenge in the developing world (2001).

(b) World Health Day: April 7th 2001 focused on mental health as its theme. Mental Health also was the focus in the World Health Assembly Meet in May 2001.

(c) World Health Report 2001 publication: the focus was on Mental Health

(d) WHO has also brought out Atlas about Mental Health Resources in the World, 2001.

At the same time, the unfortunate death of 31 inmates of an informal care center at Erwady in Tamil Nadu in August 2001 was at gross variance with all the international and national developments, which had been positively directed. The public outcry was loud and the Hon'ble Supreme Court also took notice of the incident and its possible causes by initiating the Public Interest Litigation (PIL) in 2001, which got linked up with another PIL by the NGO Saarthak, which also showed concern for some similar issues of provision of services and some other human rights issues like the use of physical restraint and use of direct ECT. The Hon'ble Supreme Court is continuing it's monitoring of the implementation of the MHA, 1987 and the adequacy of mental health services in different parts of the country. The Indian Psychiatric Society has, one more time, demonstrated its proactive interest in matters of public policy by "impleading" in these ongoing PILs, which are likely to have far reaching implications for the mental health services in the next few decades. It should be noted that the tragedy at Erwady occurred due to the non-monitoring of the unorganized sector as well as the public attitudes, which point at our inadequacy in taking the modern scientific knowledge to them. One offshoot of this was the survey of mental hospitals/state psychiatric hospitals carried out in Dec. 2001 on the orders of Hon'ble Supreme Court, which has led to specific provisions for funds for upgrading the services in the hospital sector. Money is provided for improvement of all mental hospitals in the tenth plan. The DMHP, which had a humble beginning, is to spread to 100 districts of the country. 190 crore rupees have been earmarked in the tenth plan. Three centers: CIP Ranchi, IHBAS Delhi and NIMHANS Bangalore, are to be the resource centers and will be monitoring and evaluating the Programme implementation.

It is interesting that the much-awaited Mental Health Act of 1987, which was expected to bring about significant improvements in the quality of services, has indeed become a matter of intense debate. It is also worth noting that the provisions of the MHA, 1987 and the activities of the National Mental Health Programme can be seen to be at variance with each other. It can be argued that the lack of a well thought out and clear policy on mental health contributes to such divergence in the statutory legal provisions and the programmes of the Govt. The logical connectivity of Policy to Programme to Legislation seems to have been ignored in the mental health scenario of India.

I would like to point out here that not only many of us in the general public and the concerned profession, but even official agencies seem to lose sight of the important difference between policy & programme. It is worth pointing out that although India has a National Mental Health Programme but no policy, the Atlas of Mental Health by the WHO headquarters shows us both the policy and the programme being available in India. The need for a clear Mental Health Policy has also been recognized and being recommended by the WHO Headquarters. One of the results of the World Health Report (WHR), 2001 that focused on Mental Health has been the Mental Health Policy Project by the WHO, which is aimed at assisting countries in formulation and development of mental health policy based on some important parameters. The modules being developed by the WHO for helping policy formulation are (1) The Mental Health Context, (2) Mental Health Policies & Plans, (3) Financing, (4) Legislation & Human Rights, (5) Role of Advocacy in National Planning, (6) Quality Improvement for Stewardship, (7) Organization of Services, (8) Planning & Budgeting for Service Delivery, and (9) Quality Improvement for Service Delivery. The expectation is that policy formulation and the consequent programme & legislation will help in achieving the goal of fulfilling the mental health service gap, as the Global Action Programme of the WHO has proposed by the abbreviation "mhGAP".

Whereas, it is heartening to note that India has been in the forefront in some of these events at the international level, there is a great need to address the issues at home .We should also take initiative in terms of bringing out an atlas of our country, state wise indicating the facilities and resources available, so that disparities can be seen at a glance and hopefully those issues can then be addressed. Whereas, UK has felt the need that they must enhance their manpower in psychiatry by recognizing our Indian degrees and experience and offering them lucrative terms; we have been depleted of the already meagre number of professionals in the process. We have not been even able to improve upon our policy on mental health and implement it in terms of enhancing our training at undergraduate level. There is a sort of schizophrenia that exists in us. Central Council of Health, the highest policy laying body in Health, has time and again emphasized this need but Medical Council of India has shied away from their responsibility. Even current training programs of doctors and other workers has been (hopefully temporarily?) suspended! This incidentally was an essential component (all through) of Mental Health Program.

NMHP & DMHP both lack an urban perspective. Over the period, a strong need has been felt in some quarters that urban mental health problems are unique and cannot be ignored any longer. ICMR-WHO had carried out a series of workshops and has now initiated a multi-site study starting with assessment of needs and existing facilities and resources. Issues regarding urban mental health may also address the mental health problems in homeless population, slum dwellers, the street children and other groups like adolescents and the elderly, issues of women's mental health—particularly those subjected to domestic violence perpetrated by those men affected with Alcohol/Drug Abuse. This needs to be rectified in the formulation of the new mental health program.

Recently, 'Common Mental Disorders' (CMD) have been increasingly the focus of attention. They are illnesses, which present with medically unexplained physical symptoms, depression and anxiety. They are commonest of all disorders in primary or general health care settings. CMD are amongst the most significant cause of disability in the world. About 1 in 3 adults attending primary or general heath care facility suffer from clinically significant CMD. Women and those who are less educated or are suffering acute financial difficulties are at greater risk. Gender and poverty related factors play a key role in the risk for CMD and they are also important issues with specific relevance to policies directed towards health of the poor and women's health. Thus, it has been rightly argued that CMD should be integrated at all levels of health programs directed at these vulnerable groups of the population. Our training programs for doctors and other health workers need to focus on such relevant issues so as to detect and treat CMD early enough.

Disaster and mental health is another very important area which needs to be addressed. ICMR has pioneered research in this area since the time of Bhopal Disaster in 1984. This has been followed up with study of health consequences of Marathwada earthquake disaster and recently the Gujarat earthquake study. There have also been population groups, which have been subjected to terrorist violence in some of the states. Earlier Punjab and now the State of J&K have been particularly affected. Some of the North –Eastern states like Assam and other Border States are more prone. There is a need to develop models of mental health care and evaluate the effectiveness of utilizing the community resources like the affected population, family members, community volunteers, developmental workers and primary healthcare personnel. Working closely with these the research team also learnt a great deal about the inner strength and the resilience to withstand such stresses. Infact there in may lie a key for the promotive aspects of positive mental health. There is also close need to study and educate the media about the reporting of such events, as they are very sensitive issues. Govt. of India is currently formulating a national disaster policy in which Mental Health component needs to be integrated. Thus NMHP will have to think of the needs of these states at a different level. Govt. of India is particularly aware of their need for special attention to these states and such issues and funding may not be much of a problem.

Talking of funds, apart from the Govt. and international agencies other sources may have to be tapped. So far, in the Ministry of Health the (unwritten) rule was that Govt. dealt with Govt. of India departments in other states and/or other international agencies. As private sector is fast emerging as a major player, govt. may like to consider funding NGO's whose credibility is not in doubt. For example, during my tenure, we did recommend center at Wardha for WHO funding. Similarly, raising funds through philanthropic organizations and individual donors may and should be considered. Novel methods like music concerts by celebrities may be other way of raising funds and utilizing for the mentally ill. The much talked about stigma may also be thus tackled effectively through a possible change of attitude when the issues regarding mental illness are brought into focus and limelight. Electronic media apart from print media has already been doing commendable work in spreading correct knowledge and dispelling myths and misconceptions. The role of radio and local newspapers should not be forgotten. Indian Psychiatric Society as well as its state branches can play a major role in this endeavour.

Another role for our society may be to get involved in public education through lectures or interactive symposiums to some target groups-say school /college students, teachers, police, judiciary, etc. through its state branches. So far the policy of the Govt of India as well as state govt. has been to involve only govt. aided or funded institutions and organizations to run the program. There needs to be a shift in this policy. Our society and other national association (IAPP) may play a more active role. The involvement of the President of our society in meetings of the central mental health authority, already started, should be legally incorporated in the Mental Health Authority Rules. Likewise, the state level meetings may include president of the branches of IPS. A pro-active role for the society is suggested. Herein, I may draw your attention to the role played by the American Psychiatric Association in formulating national policies and implementing them.

My approach in this oration has been more of an experiential one. Emic approach is well recognized in Psychiatry. Psychiatry is obsessed with evidence based on mental health and statistics, forgetting the crucial importance of emic approach. Young psychiatrists to be, need to have good role models around them. Books and journals alone can not teach you psychiatry because psychiatry is as much an art as it is a science.

Talking in the same vein, I have been feeling for some time that Indian Psychiatric Society may undertake task of bringing into light through the biographic account of some of the luminaries in psychiatry, their work and contribution and their thoughts, also highlighting their experiences and the difficult circumstances under which they were working so that younger generation can learn from them. This will also mean writing history of Indian psychiatry in its true perspective. For example, how I wish we should have compiled and preserved in the library the lectures of Prof. N.C. Surya, which were very original and thought provoking. While writing this oration, I tried to collect information about Prof. D.L.N. Murthy Rao and could get it only in bits and pieces. He was a great teacher and visionary apart from being Director, NIMHANS who was snatched away by cruel fate quite early in his career. I pay my respect to him and pray that all of us should emulate him.

### REFERENCES

Agarwal P, Bhatia MS, Malik SC. (1990) : "Postpartum Psychosis: a study of indoor cases in a general hospital psychiatric clinic" Acta Psychiatr. Scand. 81: 571-575.

Atlas (2001) : Mental Health Resources in the World: Mental Health Determinants and Population, Department of Mental Health and Substance Dependence, Geneva, WHO.

Bower P, Sibbald B. (2000) : Systematic review of the effect of on-site mental health professionals on the clinical behaviour of general practitioners, BMJ, 320 : 614-617.

Central Council of Health. Resolutions on Mental Health Programme, 1995,1999,2001.

Chisholm D, Sekar K Kishore Kumar K, Saeed K, James S, Mubbashar M, Murthy RS. (2000) : Intergration of mental health care into primary care; Demonstration Cost - Outcome Study in India and Pakistan, British Journal of Psychiatry. 174: 581-58.

Desai NG, Shah B, Singh RA (2003) : Current Situation Analysis of Urban Mental Service in India. Report of WHO-ICMR Workshops, Delhi.

Desai NG, Shah B. (2002) : Approach paper on workshop on Urban Mental Health (ICMR-WHO Project). Presented at the regional workshops at Mumbai, Chennai, Lucknow & Ranchi and National Workshop at New Delhi.

DGHS (1982) National Mental Health Programme for India. DGHS, New Delhi, Govt. of India.

Goldberg D, Gater R. (1996) Implications of the World Health Organization Study of Mental Illness in General Health Care for Training Primary care staff. British Journal of General Practice. 46: 483-485.

Improving Women's Health in India: Development in Practice. (1996) : World Bank, Washington D.C., U.S.A.

Issac MK. (1988) : Collaborative study on severe mental morbidity. ICMR Bulletin: New Delhi, Vol.18, No.12.

Malik S.C. (1993) "Women and Mental Health", Presidential Address delivered at ANCIPS, Lucknow.

Malik SC. (2001), "Consultation Liaison Psychiatry—Past, Present &Future" Inaugural Address, Annual Conference, North Zone, IPS, Bikaner, Rajasthan.

Malik SC. (2001). Inaugural Address, WHO workshop on Alcohol &Drug Addiction, Wardha, Maharashtra.

Malik SC. (1998), "Social Psychiatry: A Perspective", Presidential Address, Annual Conference Indian Association of Social Psychiatry Bhubaneshwar, Orissa

Mental Health Policy Project (2001) : Department of Mental Health and Substance Dependence, Geneva, WHO.

mhGAP (2002) : mental health Global Action Programme, Department of Mental Health and Substance Dependence, Noncommunicable Diseases and Mental Health ,Geneva ,WHO

Patel V, Chisholm D, Rabe-Hesketh S, Dias-Saxena F, Andrew G, Mann A. (2003) : Efficacy and cost –effectiveness of a drug and psychological treatment for common mental disorders in general health care in Goa, India. Lancet. 361: 33-39.

Patel V, Pereira J, Coutinho L, Fernandes R, Fernandes J, Mann A. (1998): Poverty, Psychological Disorder & Disability in Primary Care Attenders in Goa, India. British Journal of Psychiatry. 171: 533-536.

Patel V, Pereira J, Coutinho L, Fernandes R. (1997) : Is the labeling of Common mental disorders as psychiatric illness useful in primary care? Indian Journal of Psychiatry, 39: 239-246.

Patel V, Pereira J, Mann A. (1998) : Somatic and Psychological Models of Common Mental Disorders in India. Psychological Medicine. 28: 135-143.

Patel V. (1999) : The epidemiology of Common Mental Disorders in South Asia. NIMHANS Journal, 17: 307-327.

Poverty and Health, Regional Issues: South-East Asia, (1997), WHO, Regional Office for South –East Asia, New Delhi

Psychosocial and Mental Health Aspects of Women's Health (1993), Division of Family Health, Division of Mental Health, Geneva, WHO

R. Gater, B. Dalmeida E Sousa, G. Barrientos, J. Caraveo, C.R. Chandrashekar, M. Dhadpale, D. Goldberg. A.H. Al Kathiri, M.Mubbashar, K.Silhan, D.Thong, F.Torres-Gonzales and N.Sartorius (1991): The Pathways to Psychiatric Care: a Cross- Cultural Study. Psychological Medicine, 21, 761-774.

R. Srinivasa Murthy. & Barbara J. Burns. (1992) : Editors Proceedings of the Indo-US Symposium on Community Mental Health, NIMHANS Publication No.29, Bangalore, India

R. Srinivasa Murthy. (1988) Editor : Community Mental Health News. Issues nos. 11 & 12. ICMR Center for Advanced Research on Community Mental Health NIMHANS, Bangalore, India

Report on National Workshop on Mental Health Care for State Health Administrators (1996) : NIMHANS, Bangalore, India

Sartorius N (1989) : The World Health Organization Views on the prevention of mental disorders in developed and developing countries, in Epidemiology and the Prevention of Mental Disorders Edited by Cooper B, Helgason T.New York, Roultedge, pp 321-326.

Sartorius N, Henderson A. (1992) : The neglect of Prevention in Psychiatry, Aust. NZ J Psychiatry ; 16: 550-553.

Sartorius N. (1997) : Psychiatry in the Framework of Primary Health Care: A Threat or Boost to Psychiatry? Am. J. Psychiatry Festschrift Supplement, 154: 667-72.

Tevfik Bedirhan Ustun. (1994) : WHO Collaborative Study: an epidemiological survey of psychological problems in general health care in 15 centers worldwide. International Review of Psychiatry. 6, 357-363.

The World Health Report (1987) : Mental Health: New Understanding, New Hope, Geneva, WHO

Varma V.K., Kulhara P., Masserman C.M., Malhotra A., Malik S.C. (1998): Editors : Social Psychiatry- A global perspective , Macmillan India Ltd., New Delhi.

Vision Document on Mental Health (2001) : ICMR, New Delhi.

World Health Organization, (1992) : Mental Health Programmes, Concepts and Principles, Geneva, WHO.

World Health Organization: Primary Health Care, (1978) : Report of the International Conference on Primary Health Care, Alma Ata, 6-12 Sept., Geneva, WHO
